# The Golgi Apparatus May Be a Potential Therapeutic Target for Apoptosis-Related Neurological Diseases

**DOI:** 10.3389/fcell.2020.00830

**Published:** 2020-08-31

**Authors:** Qiang He, Hui Liu, Shuwen Deng, Xiqian Chen, Dong Li, Xuan Jiang, Wenbo Zeng, Wei Lu

**Affiliations:** ^1^Department of Neurology, The Second Xiangya Hospital, Central South University, Changsha, China; ^2^Department of Neurology, Xiangya Hospital, Central South University, Changsha, China; ^3^State Key Laboratory of Virology, CAS Center for Excellence in Brain Science and Intelligence Technology, Wuhan Institute of Virology, Chinese Academy of Sciences, Wuhan, China

**Keywords:** Golgi apparatus, apoptosis, caspases, neurological diseases, therapeutic target

## Abstract

Increasing evidence shows that, in addition to the classical function of protein processing and transport, the Golgi apparatus (GA) is also involved in apoptosis, one of the most common forms of cell death. The structure and the function of the GA is damaged during apoptosis. However, the specific effect of the GA on the apoptosis process is unclear; it may be involved in initiating or promoting apoptosis, or it may inhibit apoptosis. Golgi-related apoptosis is associated with a variety of neurological diseases including glioma, Alzheimer’s disease (AD), Parkinson’s disease (PD), and ischemic stroke. This review summarizes the changes and the possible mechanisms of Golgi structure and function during apoptosis. In addition, we also explore the possible mechanisms by which the GA regulates apoptosis and summarize the potential relationship between the Golgi and certain neurological diseases from the perspective of apoptosis. Elucidation of the interaction between the GA and apoptosis broadens our understanding of the pathological mechanisms of neurological diseases and provides new research directions for the treatment of these diseases. Therefore, we propose that the GA may be a potential therapeutic target for apoptosis-related neurological diseases.

## The Golgi Apparatus

In plants, invertebrates, and many protists, Golgi stacks are independent of each other and scattered throughout the cytoplasm. In vertebrates, Golgi mini-stacks are laterally fused into a twisted continuous ribbon structure that is maintained at the centrosome and paranuclear position by interaction with microtubules and dynein motors (Makhoul et al., 2018; Kulkarni-Gosavi et al., 2019). This difference obviously reveals that the higher complexity of the Golgi apparatus (GA) structure is required for more complex functions in vertebrates, especially mammals. The GA lies at the heart of the secretory pathway; proteins and lipids are sequentially modified and processed in the Golgi stacks to ultimately help target transports to their correct destination (Lowe, 2011). In addition to these classic functions, the GA is also involved in higher-order functions such as mitosis (Lowe et al., 2000), DNA repair (Farber-Katz et al., 2014), stress response (Li T. et al., 2016), autophagy (Chang et al., 2012), inflammation (Chen and Chen, 2018), and apoptosis (Chiu et al., 2002). Furthermore, the GA is an important signaling platform for many cascade signals originating from the plasma membrane or other organelles, including ubiquitin ligases, phospholipases, phosphatases, and many types of trimeric G proteins (Mayinger, 2011).

## Apoptosis

Apoptosis is an orderly autonomous death process controlled by genes. In this process, cells that are harmful to the body or unneeded will be removed (Peter, 2011). If infected cells are apoptotic before viral progeny are produced, virus replication will be limited (Nguyen and Blaho, 2006). In ischemic stroke, apoptosis is a vital pathway that mediates neuron death (Xu et al., 2019). Three main apoptotic pathways are known: receptor-mediated apoptotic pathways, mitochondria-mediated apoptotic pathways, and endoplasmic reticulum (ER)-mediated apoptotic pathways. These apoptotic pathways will eventually be integrated into the caspase cascade pathway, where the effector caspases will cleave proteins that maintain cell structure and metabolism. Then, the apoptotic cells will be engulfed and degraded by nearby phagocytes or other surrounding cells. In receptor-mediated apoptotic pathways, the most common death receptors on the plasma membrane surface [such as Fas and tumor necrosis factor receptors (TNFRs)] bind to the corresponding ligands to transmit apoptotic signals, thereby activating the caspase cascade pathway. In mitochondria-mediated apoptotic pathways, the mitochondrial permeability transition pore is irreversibly over-opened under various proapoptotic signals, which causes the release of cytochrome C (an active proapoptotic protein) from mitochondria into the cytosol to initiate the downstream apoptotic pathway. In ER-mediated apoptotic pathways, the most common apoptotic pathway (unfolded protein response, UPR) is primarily triggered by misfolded and unfolded proteins in the ER. In UPR, CHOP, and caspase 12 play key roles in apoptotic signal transmission (Elmore, 2007; Savitskaya and Onishchenko, 2015). In fact, according to the source of the apoptosis-stimulating signal, the mitochondria- and the ER-mediated apoptosis pathways can be collectively referred to as intrinsic apoptosis pathways, and the receptor-mediated apoptosis pathways can be referred to as extrinsic apoptotic pathways. The above-mentioned apoptotic pathways are regulated by the Bcl-2 family of proteins, which is divided into anti-apoptotic proteins (such as Bcl-2 and Bcl-xL) and proapoptotic proteins (such as Bak and Bax) (Popgeorgiev et al., 2018). In addition, p53 transcription factor activation promotes the expression of proapoptotic proteins such as Bax, Puma, and NoxA. In turn, these factors induce mitochondria-mediated apoptosis and subsequent caspase cascade activation (Savitskaya and Onishchenko, 2015).

## Apoptosis Affects the GA

**FIGURE 1:**
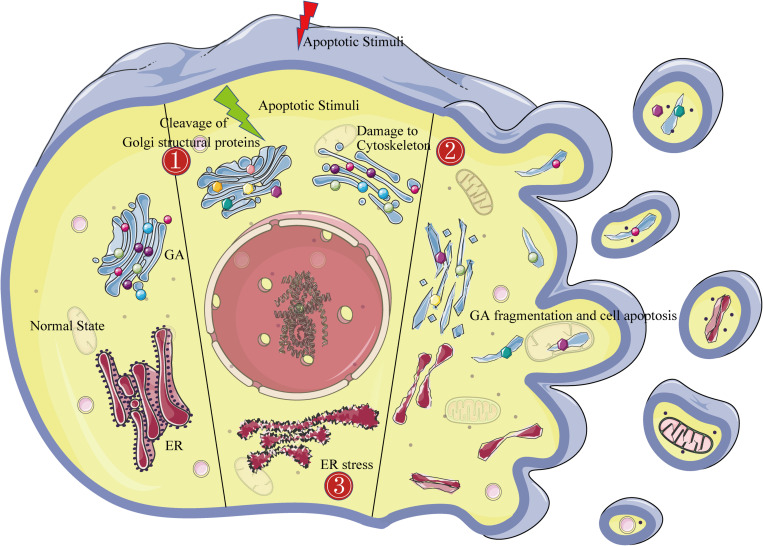
The Golgi fragmentation mechanism during apoptosis. The left side of the figure shows the morphological structure of the endoplasmic reticulum and the Golgi in normal cells. The middle of the figure shows the three mechanisms of Golgi fragmentation in apoptotic cells: ➀ activated caspases cleave Golgi structural proteins (represented by dots of various colors); ➁ the cytoskeleton is damaged (represented by blue bands); and ➂ endoplasmic reticulum stress. The right side of the figure shows the complete fragmentation of the Golgi apparatus at the end of apoptosis.

The in-depth study of the apoptosis mechanism has led to an increasing number of technical means for detecting apoptosis. However, morphological observations play an important role in distinguishing apoptosis from other types of cell death. Of these, transmission electron microscopy is considered to be the gold standard for confirming apoptosis (White and Cinti, 2004). Apoptotic cell characteristics that can be observed by transmission electron microscopy include the following: (1) an electron-dense nucleus (marginalization in the early phase), (2) nuclear fragmentation, (3) an intact cell membrane even late in the cell disintegration phase, (4) disorganized cytoplasmic organelles, (5) large clear vacuoles, and (6) blebs at the cell surface. Cells will break into apoptotic bodies with intact plasma membranes that contain cytoplasmic organelles in the later stages of apoptosis (Elmore, 2007). Obviously, Golgi morphology also changes drastically during apoptosis. In normal cells viewed by transmission electron microscopy, the GA pattern is initially highly ordered, appearing as ribbon-like stacks in a vesicular structure. In cells treated with apoptosis inducers, the Golgi stacks become swollen, disordered, and lysed into vesicles and tubules (Snigirevskaia et al., 2014). Similarly, Golgi marker immunofluorescence shows that the GA pattern is dispersed and fragmented throughout the cytoplasm in apoptotic cells, unlike in normal cells where it accumulates in the perinuclear area in a typical semilunar shape (Bottone et al., 2013). While morphological alterations of the GA are common in various physiological or pathological conditions, the Golgi fragmentation mechanism in apoptosis is quite distinct (Figure 1).

### Activated Caspases Cleave Golgi Structural Proteins

One of the characteristics of apoptosis is caspase cascade signaling activation including caspase 3, caspase 8, or caspase 9 (Savitskaya and Onishchenko, 2015). Activated caspases cleave important structural proteins and cause the destruction of organelles, such as the Golgi (Chiu et al., 2002), mitochondria (Yu et al., 2014), and nucleus (Al-Ghorbani et al., 2016). The currently known Golgi structural proteins that can be cleaved by activated caspases are described in the following subsections.

#### GM130

One of the most widely studied Golgi matrix proteins is GM130, which is anchored to the *cis-*Golgi. An important step in the biogenesis of the Golgi ribbon is to dynamically and continuously incorporate vesicles from the ER into the Golgi stacks (Marra et al., 2007). GM130 can specifically capture vesicles from the ER (Wong and Munro, 2014; Lowe, 2019). If GM130 is knocked out, this capture process will be ablated, resulting in the shortening of the Golgi cisternae and the disruption of the Golgi ribbon (Marra et al., 2007; Liu et al., 2017). Recent studies have shown that the methylation of the N-terminal arginine of GM130 plays an important role in maintaining the structure of the Golgi (Zhou et al., 2010). During Fas-induced apoptosis, GM130 decreases rapidly, accompanied by GA fragmentation (Walker et al., 2004). He et al. (2020) found that HSV-1 infection-induced endothelial cell apoptosis and Golgi fragmentation were significantly reduced after GM130 cleavage was inhibited by the pan-caspase inhibitor, Z-VAD. These results reveal that activated caspases cleave GM130, which might be involved in GA disruption during apoptosis.

#### Giantin

Giantin, primarily localized to the Golgi cisternal rims, is the largest golgin in mammals (Linstedt et al., 1995). Koreishi et al. (2013) found that giantin plays a role in laterally linking the Golgi cisternae within the Golgi ribbon. Depletion of giantin with siRNA causes dispersion of the Golgi stacks. However, the exogenous expression of giantin in *Drosophila* S2 cells promotes Golgi stack clustering, similar to the Golgi ribbon in mammalian cells (Koreishi et al., 2013). During apoptosis, giantin is cleaved by caspase 2, caspase 3, and caspase 7, accompanied by the cessation of vesicular transport between the ER and the GA (Lowe et al., 2004). We speculate that cleavage of giantin by activated caspases could promote fragmentation of the Golgi.

#### P115

P115 consists of an N-terminal globular head, a coiled-coil tail, and a short C-terminal acidic domain, often forming dimers which are found in the *cis-*Golgi (An et al., 2009). It was initially found that P115 mainly acts on intercisternal transport in the Golgi stacks (Waters et al., 1992). Both GM130 and giantin can bind to the C-terminal acidic tail of P115. Further experiments confirmed that P115, GM130, and giantin form a tethering complex, tethering coat protein complex I vesicles to the Golgi membranes (Sönnichsen et al., 1998; Beard et al., 2005). Mice lacking P115 exhibit destruction of the Golgi structure, revealing the importance of P115 in maintaining the integrity of the Golgi (Kim et al., 2012). A recent experiment found that P115 is cleaved by caspase 3 and caspase 8 during apoptosis (Chiu et al., 2002). Relative to that, in control cells expressing wild-type P115, cells expressing the cleavage-resistant form of P115 show delayed Golgi fragmentation during apoptosis. These experimental results revealed that Golgi fragmentation during apoptosis is partly due to P115 cleavage (Chiu et al., 2002).

#### GRASP65

GRASP65 is mainly targeted to the *cis-*Golgi and is a member of the Golgi reassembly and stacking protein (GRASP) family (Zhang and Wang, 2015). However, whether GRASP65 acts as a stacking factor is still controversial. This is because, in mammalian cells, there are contradictory reports on the integrity of the Golgi stack after the depletion of GRASP65 (Xiang and Wang, 2010; Rabouille and Linstedt, 2016). A less controversial role of GRASP65 is that it can promote formation of the Golgi ribbons in mammals. Jarvela et al. (2014) found that the inactivation of GRASP65 specifically causes the *cis-*side of the Golgi ribbons to be disconnected. In addition, Cheng et al. (2010) demonstrated that Golgi fragmentation is dependent on caspases and identified GRASP65 as a new substrate for caspase 3. The expression of the caspase-resistant form of GRASP65 partially inhibits Golgi fragmentation in apoptotic cells (Lane et al., 2002).

#### Golgin160

Golgin160 (also known as GOLGA3) is a coiled-coil protein enriched in the *cis-*Golgi cisternae (Hicks et al., 2006). Yadav et al. (2009, 2012) have proved that golgin160 can recruit the dynein microtubule motor protein to the GA, which is important for maintaining the positioning of the GA and the integrity of the Golgi ribbons. The loss of golgin160 in cultured cells results in Golgi positioning defect and generates dispersed ministacks (Yadav et al., 2009, 2012). During apoptosis, golgin160 is cleaved by caspase 2, caspase 3, and caspase 7 (Mancini et al., 2000). The inhibition of caspase-mediated golgin160 cleavage can prevent GA fragmentation and even apoptosis. The exogenous expression of a caspase-resistant mutant of golgin160 attenuates the kinetics of GA fragmentation, revealing that caspase-mediated cleavage of golgin160 is involved in inducing apoptotic changes in Golgi morphology (Mancini et al., 2000; Maag et al., 2005).

#### Golgin84

Golgin84 is a member of the golgin family, which is mainly presented on the Golgi cisternal rims (Gillingham and Munro, 2016). Satoh et al. (2003) found that golgin84 can stimulate Golgi stacking and increase the length of the Golgi stacks. In addition, it is generally believed that golgin84 plays an important role in tethering the intra-Golgi transport vesicles (Sohda et al., 2010; Lowe, 2019). In golgin84-knockdown cells, the Golgi ribbon breaks into ministacks, accompanied by the accumulation of some intra-Golgi transport vesicles (Sohda et al., 2010). During apoptosis triggered by *Chlamydia trachomatis* infection, activated caspases cleave golgin84, accompanied by GA fragmentation. However, inhibiting golgin84 proteolytic cleavage can prevent Golgi fragmentation (Heuer et al., 2009).

#### Syntaxin 5

Syntaxin 5, located in the *cis-*Golgi cisternae and ER, is a member of the SNARE family (Hui et al., 1997; Linders et al., 2019). It specifically pairs with other homologous SNAREs to form the “SNARE complex,” driving the fusion of the vesicle membrane with the target membrane. It has been found that syntaxin 5 is involved in the vesicle fusion events of ER–Golgi, intra-Golgi, and early/recycling endosome to the *trans-*Golgi network trafficking (Dascher et al., 1994; Tai et al., 2004; Volchuk et al., 2004). RNA interference-mediated silencing of syntaxin 5 leads to fragmentation of the GA, revealing that syntaxin 5 is necessary for maintenance of the Golgi structure (Suga et al., 2005). During apoptosis, syntaxin 5 is cleaved by caspase 3 and caspase 7, which is accompanied by disorders in vesicular trafficking (Lowe et al., 2004). We speculate that cleavage of syntaxin 5 by activated caspases is involved in Golgi fragmentation during apoptosis.

Taken together, the aforementioned Golgi proteins are either related to vesicle tethering and fusion or to Golgi stacking and ribbon formation, which are involved in maintaining the structural integrity of the Golgi. Moreover, these proteins have been confirmed to be cleaved by activated caspases during apoptosis (Table 1). Therefore, we propose that, under different apoptotic stimuli, activated caspases cleave one or several Golgi structural proteins, resulting in fragmentation of the GA.

**TABLE 1:** Caspase cleavage of Golgi structural proteins associated with Golgi fragmentation during apoptosis.

Golgi structural proteins	Golgi location	Role in maintaining the Golgi apparatus (GA) structure	Caspase-mediated cleavage
*GM130*	*cis-*Golgi	Capture the ER-to-Golgi vesicles	None identified
*Giantin*	Golgi cisternal rims	Laterally link Golgi cisternae	Caspase 2, 3, and 7 (Lowe et al., 2004)
*P115*	*cis-*Golgi	Tether coat protein complex I vesicles to the Golgi membranes	Caspase 3 and 8 (Chiu et al., 2002; Kim et al., 2012)
*GRASP65*	*cis-*Golgi	Promote the formation of the Golgi ribbons	Caspase 3 (Lane et al., 2002)
*Golgin160*	*cis-*Golgi	Maintain the positioning of GA and the integrity of the Golgi ribbons	Caspase 2, 3, and 7 (Mancini et al., 2000; Maag et al., 2005)
*Golgin84*	Golgi cisternal rims	Stimulate Golgi stacking, increase the length of the Golgi stacks, and tether the intra-Golgi transport vesicles	None identified
*Syntaxin 5*	*cis-*Golgi	Drive the fusion of vesicle membranes with the Golgi membranes	Caspase 3 and 7 (Lowe et al., 2004)

### Cytoskeleton Damage May Contribute to GA Fragmentation

The cytoskeleton mainly consists of microtubules and actin filaments that are essential for maintaining GA structure. It is generally believed that microtubules play a key role in generating and positioning the Golgi ribbon and that actin finely and synergistically regulates the Golgi architecture (Makhoul et al., 2018). Golgi-derived microtubules work in a search-and-capture manner to contact other nearby Golgi stacks. These Golgi stacks move along the microtubules toward the minus end and eventually gather near the perinuclear centrosome (Lowe, 2011). Tang et al. (2016) found that Mena interacts with GRASP65 to promote local actin polymerization, thereby promoting the interconnection between Golgi stacks to form the Golgi ribbon. In motor neurons, the overexpression of microtubule-destabilizing proteins stathmin 1 and stathmin 2 led to the defective polymerization of Golgi-derived microtubules and severe Golgi fragmentation; however, stathmin 1/2 knockdown or treatment with the microtubule-stabilizing drug taxol completely reverses this effect (Bellouze et al., 2016). Perforation/fragmentation and severe swelling of Golgi cisternae have been observed by transmission electron microscopy using actin-depolymerizing toxins (cytochalasin D, latrunculin B, mycalolide B, and *Clostridium botulinum* C2 toxin) (Lazaro-Dieguez et al., 2006). In addition, recent studies found that the formin homology 2 (FH2) domain protein 1 (FHDC1) enriched on *cis-*Golgi binds microtubules through a unique C-terminal domain and actin through its FH2 domain. Knockdown or overexpression of FHDC1 can cause the dispersal of the Golgi ribbon into mini-stacks, indicating the potential role of actin and microtubule Golgi networks in maintaining the Golgi ribbon structure (Copeland et al., 2016). Microtubules are reportedly depolymerized at apoptosis execution phase onset (Moss and Lane, 2006; Ndozangue-Touriguine et al., 2008). However, recent results indicate that the microtubule network reforms during the later stages of apoptosis and contributes to nuclear and cell fragmentation (Moss and Lane, 2006). Actin is similarly degraded during apoptosis (Coleman and Olson, 2002). Moreover, mounting evidence proves that the actin cytoskeleton is a sensor and mediator of apoptosis (Desouza et al., 2012). In addition, both tubulin and actin are cleaved by caspases (Fischer et al., 2003; Moss and Lane, 2006). Therefore, given the importance of the cytoskeleton to the GA, we speculate that cytoskeleton damage may contribute to GA fragmentation in apoptosis.

### ER Stress-Mediated GA Fragmentation

Endoplasmic reticulum and GA are closely related in morphology and function. The stability of the Golgi dynamic structure depends on efficient bidirectional vesicle transport with the ER (Kokubun et al., 2019). In a stacked Golgi, new cisternae would form at the *cis* face, pass through the stack, and then peel off from the *trans* face, carrying secretory cargo proteins forward. The vesicles of the ER would shuttle to the Golgi to form new cisternae to maintain stack integrity (Mollenhauer and Morré, 1991; Emr et al., 2009). The ER is an important organelle in eukaryotic cells that is mainly responsible for the synthesis, processing, and modification of intracellular proteins. However, unfolded and misfolded proteins can accumulate in the ER and lead to ER stress when cells are subjected to a variety of strong stimulating factors such as apoptosis inducers, toxin stimulation, or Ca^2+^ metabolic imbalance. When the stimulus persists or is too strong, ER stress activates the apoptotic pathway and disrupts the ER-to-GA transport (Short et al., 2007; Qi and Chen, 2019). The GA becomes disordered and diffused under treatment with the ER stress inducer thapsigargin (Nakagomi et al., 2008). In addition, mutant SOD1 inhibits the ER-to-GA transport of secreted proteins in neurons, thereby causing GA fragmentation (Atkin et al., 2014). Protein-disulfide isomerase Bax inhibitor 1 (BI-1), located on the ER, inhibits ER stress. Nakagomi et al. (2008) found that BI-1 overexpression in cortical neurons can partially inhibit the Golgi fragmentation induced by various insults, further revealing that ER stress plays an important role in regulating Golgi morphology. Based on these data, we propose that ER stress causes GA fragmentation to varying degrees by disrupting the ER-to-GA transport during ER stress-triggered apoptosis.

## The GA Affects Apoptosis

**FIGURE 2:**
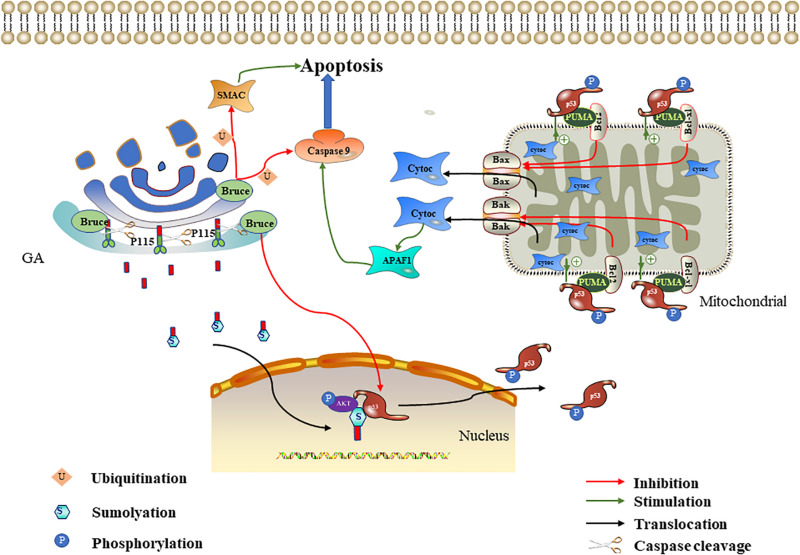
Effect of P115 and Bruce on apoptosis. The p115 caspase cleavage fragment promotes apoptosis mediated by the ERK/p53/p53 upregulated modulator of apoptosis (PUMA) pathways. The p115 caspase cleavage fragment enters the nucleus after being sumoylation and then acts as a scaffold to promote ERF-mediated p53 phosphorylation. The phosphorylated p53 upregulates PUMA. PUMA then relieves the Bcl-2/Bcl-xl inhibition of Bax/Bak, leading to mitochondrial cytochrome c release. Cytochrome c activates APAF1 and caspase 9 in succession to promote apoptosis. Bruce degrades caspase 9/SMAC through ubiquitination and downregulates p53 to inhibit apoptosis.

**FIGURE 3:**
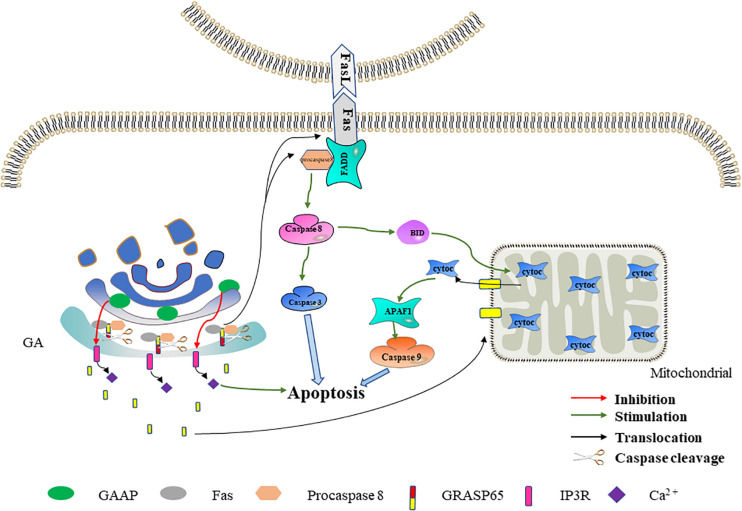
The effect of Golgi reassembly and stacking protein 65 (GRASP65) and Golgi anti-apoptotic protein (GAAP) on apoptosis. GRASP65 caspase cleavage promotes Fas-mediated apoptosis. After GRASP65 is cleaved by caspases, Golgi-tethered Fas and procaspase 8 are transported to the cell membrane, thereby further promoting apoptosis. In addition, the caspase cleavage fragment of GRASP65 will translocate to the mitochondria, thereby promoting the release of cytochrome c and promoting apoptosis. GAAP inhibits Ca^2+^ release into the cytoplasm by interacting with IP3R. The decreased Ca^2+^ in the cytoplasm inhibits apoptosis to a certain extent.

The GA is damaged by various pathways during apoptosis. However, increasing evidence shows that the subcellular organelle is involved in apoptosis signaling pathway regulation and that the GA is also involved (Hicks and Machamer, 2005; Sasaki and Yoshida, 2019). Studies have identified many components located in the GA that are related to apoptosis regulation, such as P115, GRASP65, and Bruce (Cheng et al., 2010; How and Shields, 2011; Tassi et al., 2012). A highly conserved Golgi anti-apoptotic protein (GAAP) located only in the GA has also been found (Carrara et al., 2017). The following sections focus on the related molecules and mechanisms of Golgi that promote or inhibit apoptosis signals (Figures 2, 3).

### The P115 Caspase Cleavage Fragment Promotes Apoptosis Through the ERK/p53/PUMA Pathway

P115 is a 961-kDa Golgi peripheral membrane protein. It is mainly involved in vesicle transport between Golgi cisternae (Sapperstein et al., 1995). In Chiu et al. (2002) found that P115 was cleaved by caspase 3 and caspase 8 at residue TEKD757, resulting in a C-terminal fragment (CTF) containing 205 residues during apoptosis. CTF enters the nucleus through sumoylation (Mukherjee and Shields, 2009). The CTF in the nucleus serves as a scaffold to tether ERK and p53, thereby promoting p53 phosphorylation and activation by ERK (How and Shields, 2011). ERK is a mitogen-activated protein kinase, and its signaling cascade plays an important role in apoptosis (Li et al., 2014; da Cunha Jaeger et al., 2020). p53 is an important tumor suppressor gene, and it is often involved in the transcriptional regulation of key pro-apoptotic genes, such as p53 upregulated modulator of apoptosis (*PUMA*) and *hdm2* (Khan et al., 2020). PUMA is a member of the Bcl-2 family that interacts with anti-apoptotic Bcl-2 family members (Bcl-xl and Bcl-2) and relieves the Bcl-2/Bcl-xl inhibition of Bax/Bak. When the Bax/Bak inhibition is lifted, they will translocate to the mitochondria and trigger the release of cytochrome C; this ultimately activates the caspase cascade signaling pathway and thereby induces apoptosis (Nakano and Vousden, 2001). How and Shields (2011) further proved that P115 CTF expression can promote p53 activation, thereby increasing PUMA expression. The above-mentioned literature indicates that, in the early stage of apoptosis, P115 located on the Golgi is partly cleaved to produce CTF. P115 CTF promotes the mitochondria-mediated apoptosis pathway through the ERK/p53/PUMA pathway, thereby amplifying the apoptotic signal and leading to the full activation of caspases and apoptosis (Hilton et al., 2014; Stokholm et al., 2016; Figure 2).

### Bruce Degrades Caspase 9/SMAC Through Ubiquitination and Downregulates p53 to Inhibit Apoptosis

Bruce (also known as apollon) is a conserved peripheral membrane protein located on the *trans-*Golgi. Its amino and carboxyl termini contain the baculovirus-IAP repeat (BIR) and the ubiquitin-conjugating enzymes (UBC) domain, respectively (Hauser et al., 1998). Bruce is a member of the IAP family, so named for their ability to bind and inhibit caspases and other pro-apoptotic factors (Pohl and Jentsch, 2008; Vasudevan and Ryoo, 2015). In 293T and HeLa cells, overexpressed Bruce can inhibit apoptosis induced by various stimuli, including staurosporine and ultraviolet light (Bartke et al., 2004). Bruce can inhibit apoptosis in multiple ways, and its inhibitory effect mainly plays a role in the mitochondria-mediated apoptosis pathway. On the one hand, the BIR motif on Bruce binds to activated caspase 9 and SMAC, and then the UBC domain is used to induce the ubiquitination of the two, thereby inhibiting apoptosis. Because the mitochondria release pro-apoptotic proteins SMAC and cytochrome c after mitochondria-mediated apoptosis is triggered, cytochrome c combines with Apaf-1 and dATP to form the apoptosome (a multi-protein platform with a catalytic effect). It can recruit and activate caspase 9, leading to caspase cascade signaling pathway activation (Zhang, 2019; Li et al., 2020). Therefore, the Golgi can degrade caspase 9 and SMAC through Bruce to inhibit apoptosis. Interestingly, Bruce can also be cleaved by activated caspase 9 or degraded by SMAC through the ubiquitination pathway (Hao et al., 2004; Qiu and Goldberg, 2005). Therefore, the degree of apoptosis depends on the relative activities of Bruce, caspase 9, and SMAC. On the other hand, the Golgi can inhibit apoptosis by downregulating p53 through Bruce. Ren et al. (2005) found that the deletion of the C-terminal half of Bruce activates p53 in mice, which in turn upregulates bax and bak and leads to mitochondria-mediated apoptosis. Therefore, we speculate that, under normal physiological conditions, complete Bruce should inhibit p53 expression, thereby down-regulating bax and bak and inhibiting mitochondria-mediated apoptosis. In conclusion, the Golgi can degrade caspase 9/SMAC and downregulate p53 through Bruce to inhibit apoptosis (Figure 2).

### GRASP65 Caspase Cleavage Promotes Fas-Mediated Apoptosis

Fas (also known as CD95) is a member of the TNFR superfamily, which transmits apoptotic signals by binding to Fas ligand (FasL) expressed in the cell membranes of other cells (Andera, 2009; Xie et al., 2019). The Fas–FasL system is a major signaling pathway for apoptosis induction. After binding to FasL, the Fas receptor interacts with the signal adapter, FADD, through its death domain (Thomas et al., 2004). FADD then recruits inactive procaspase 8 through a homologous motif. Fas, FADD, and procaspase 8 form a death-inducing signaling complex (Gomez-Angelats and Cidlowski, 2001). After the complex is formed, procaspase 8 drives its activation through self-cleavage. The activated caspase 8 directly activates the caspase cascade signaling pathway or cleaves BID to trigger the mitochondria-mediated apoptosis pathway to indirectly activate the caspase cascade signaling pathway, thereby leading to apoptosis (El Mchichi et al., 2007; Logue and Martin, 2008).

GRASP65 is a *cis-*Golgi protein that plays a role in Golgi structure, membrane trafficking, and cell signaling (Ahat et al., 2019a, b). In the early stage of apoptosis, cleavage of GRASP65 by caspase 3 may promote Fas-mediated apoptosis in one of two ways (Lane et al., 2002). In one method, the Golgi promote the translocation of Fas and procaspase 8 to the cell membrane by GRASP65 cleavage, thereby promoting Fas-mediated apoptosis. GRASP65 can regulate the transport of proteins containing C-terminal hydrophobic motifs on the GA through its tandem PDZ-type “GRASP” domains. The Fas C-terminus terminates with a hydrophobic leucine–valine motif (Ivanov et al., 2003). The interaction of GRASP65 with Fas and procaspase 8 has been detected in isolated Golgi components during hypoxia/reoxygenation-induced apoptosis (Wang et al., 2004). Therefore, we speculate that, in the early stage of apoptosis, GRASP65 tethers Fas and procaspase 8 to the GA through its PDZ domain. When GRASP65 is cleaved by caspase 3, Fas and procaspase 8 are transported to the cell membrane to promote apoptosis. In the other method, the GA releases the GRASP65 caspase fragment to the mitochondria, thereby promoting Fas-mediated apoptosis. Cheng et al. (2010) found that the C-terminus obtained by cleaving GRASP65 targets the mitochondria during Fas-mediated apoptosis. In addition, the GRASP65 caspase cleavage C-terminus fragment can make cells more sensitive to apoptosis induced by mitochondrial toxicants such as CCCP, antimycin A, and FasL (Cheng et al., 2010). These results reveal that, following the mitochondrial targeting of the caspase cleavage fragment released by GRASP65, mitochondrion permeability increases and thereby promotes cytochrome c release and apoptosis. Based on the above-mentioned data, we speculate that the Golgi may further promote Fas-mediated apoptosis through GRASP65 caspase cleavage (Figure 3).

### GAAP Inhibits Apoptosis by Reducing Cytosolic Ca^2+^ Flux

A new anti-apoptotic protein has been isolated and identified in camel pox virus. It was named GAAP based on its intracellular localization and its first described function. GAAP has significant amino acid conservation with orthologs throughout eukaryotes, prokaryotes, and some orthopoxviruses, indicating a high degree of conserved functions (Gubser et al., 2007). In addition to its anti-apoptotic functions, GAAP also regulates the Ca^2+^ flux of the GA and ER and forms cation-selective channels. GAAP is widely expressed in various tissues of the human body (Gubser et al., 2007; de Mattia et al., 2009; Carrara et al., 2017).

Ca^2+^ is a ubiquitous intracellular messenger involved in both intrinsic and extrinsic apoptosis signaling pathways (Tiwari et al., 2017; Zhou et al., 2019). Under physiological conditions, Ca^2+^ in the cytoplasm is maintained at a low level, while subcellular organelles such as ER and GA store higher concentrations of Ca^2+^. Ca^2+^-induced signals are triggered by its transmembrane entry and/or release from the cell reservoir (mainly the ER and the GA) (Chung et al., 2017; Smaardijk et al., 2017). Ca^2+^ in the ER and the GA is mainly released through the IP3R (inositol-1,4,5-trisphosphate receptor) channel (Lin et al., 1999; Parys and Vervliet, 2020). The Ca^2+^ signals between Ca^2+^ storage organelles and mitochondria play an important role in promoting apoptosis (Marchi et al., 2018). Molecular and pharmacological methods that reduce cytoplasmic Ca^2+^ levels can protect cells from apoptosis, while conditions that increase cytoplasmic Ca^2+^ levels have the opposite effect (Ma et al., 1999; Nakamura et al., 2000; Pinton and Rizzuto, 2006). GAAP inhibits extracellular Ca^2+^ influx and reduces Ca^2+^ release from organelle reservoirs by interacting with IP3R, thereby reducing the increase in cytosolic Ca^2+^ flux induced by apoptotic stimuli (de Mattia et al., 2009). Therefore, we speculate that the Golgi may reduce the cytosolic Ca^2+^ flux and inhibit apoptosis through GAAP. In addition, Guo et al. (2018) found that GAAP in *Arabidopsis* interacts with IRE1, which initiates ER stress and thereby inhibits ER stress-induced apoptosis. The association between GAAP and ER stress in *Arabidopsis* provides a direction for future research on the human GAAP anti-apoptosis mechanism (Figure 3).

## Golgi-Related Apoptosis in Neurological Diseases

**FIGURE 4:**
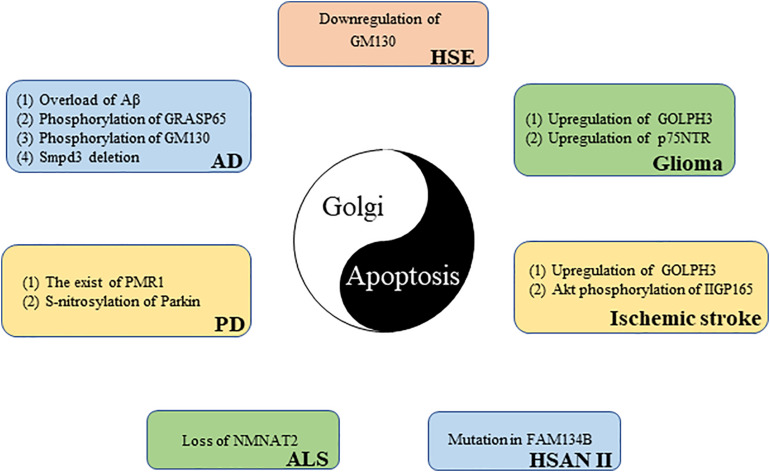
The link between the Golgi apparatus and neurological diseases from the perspective of apoptosis.

Apoptosis is an important pathological mechanism in many neurological diseases (Su et al., 2019), and the GA can perceive and transmit apoptotic signals through its own unique molecular mechanism (Machamer, 2015). More than 40% of GA-related genes known to be associated with disease affect the central or the peripheral nervous system, highlighting the importance of the GA in neurological diseases (Zappa et al., 2018). The following discussion explores the potential link between Golgi and certain neurological diseases from the perspective of apoptosis (Figure 4).

### Glioma

Neuroepithelial-derived tumors are collectively called gliomas; they account for 30% of all brain and central nervous system tumors and 80% of all malignant brain tumors (Goodenberger and Jenkins, 2012). GOLPH3, a member of the *trans-*Golgi network protein family, is the first example of an oncogene that functions in secretory trafficking at the GA (Buschman et al., 2015). GOLPH3 is highly expressed in glioma tissues, and its expression level is directly proportional to tumor malignancy. However, there is a negative correlation between tumor suppressor gene NDRG1 expression and GOLPH3 expression in glioma samples. GOLPH3 knockdown increases NDRG1 and triggers apoptosis in glioma cells. These results reveal that GOLPH3 causes glioma cells to escape apoptosis monitoring by downregulating NDRG1, which may promote glioma occurrence and development (Li X. et al., 2016). The poor prognosis of glioma patients is largely due to acquired chemotherapy resistance. Peng et al. (2018) found that GOLPH3 regulation sensitizes glioma cells to apoptotic stimuli induced by the chemotherapy drug temozolomide. The p75 neurotrophin receptor (p75NTR) is a glycoprotein belonging to the TNFR/nerve growth factor receptor family that is traditionally detected at the cell membrane (Wang et al., 2020). Recently, Giraud et al. (2011) found, using immunofluorescence microscopy, that a part of p75NTR is located in the GA of glioma cells. p75NTR retention in the GA prevents p75NTR expression on the cell surface. p75NTR can be sequestered in the GA; this sequestration is responsible for cell resistance to apoptosis and glioma formation (Giraud et al., 2011).

### Alzheimer’s Disease

Alzheimer’s disease (AD) patients show a progressive decline in memory and cognitive function because AD leads to the gradual loss of neuronal function (Bondi et al., 2017). One of the main pathological features of AD is the abnormal production and deposition of amyloid beta peptide (Aβ). Aβ is mainly produced in the *cis-*Golgi (Choy et al., 2012). However, too much Aβ can cause GA fragmentation before cell death. Experiments revealed that Golgi fragmentation in AD is caused by GRASP65 phosphorylation, which is induced by Aβ-triggered cyclin-dependent kinase 5 (Cdk5) activation (Joshi et al., 2014). GM130 is also phosphorylated by Aβ-activated Cdk5, causing GA fragmentation (Sun et al., 2008). Golgi fragmentation, in turn, promotes Aβ production (Joshi et al., 2014). The aggregated Aβ neurotoxicity manifests as neuronal apoptosis, in which caspase 8, caspase 9, and caspase 3 are activated (Takada et al., 2020). These results reveal the importance of the Golgi in AD (Joshi et al., 2014). In addition, Stoffel et al. (2018) found that neutral sphingomyelinase, smpd3, expression in the central nervous system is restricted to the GA of neurons. In the brains of smpd3−/− mice, Golgi vesicular protein transport in neurons is inhibited, which leads to Aβ deposition, UPR, and apoptosis. Finally, smpd3−/− mice show a progressive cognitive decline similar to the clinical manifestations of familial and sporadic AD, indicating that smpd3 may be a susceptible gene for AD (Stoffel et al., 2018).

### Amyotrophic Lateral Sclerosis

Amyotrophic lateral sclerosis (ALS) is a deadly neurodegenerative disease characterized by the progressive degeneration of motor neurons (Zhang et al., 2019). Nicotinamide adenine dinucleotide (NAD^+^) is an important cofactor for cell metabolism maintenance. By increasing NAD^+^ expression, survival time is moderately prolonged in ALS mice (Harlan et al., 2020). NMNAT2, a key enzyme responsible for NAD^+^ production, is mainly located in the GA (Milde et al., 2013). Harlan et al. (2020) found that the NMNAT2 expression is reduced in the spinal cord of ALS patients. The loss of NMNAT2 in neurons can cause GA fragmentation, further leading to neuronal apoptosis (Pottorf et al., 2018). These results suggest that NMNAT2 may be a potential target for the treatment of ALS.

### Parkinson’s Disease

Parkinson’s disease (PD) is a degenerative neurological disease commonly found in middle-aged and elderly people that it is characterized by a progressive loss of dopaminergic neurons (Surmeier, 2018). The main toxic effector of PD is α-synuclein, which destroys intracellular Ca^2+^ homeostasis and causes neuronal death. Buttner et al. (2013) demonstrated that the Ca^2+^/Mn^2+^ ATPase, PMR1, residing on the GA is a conserved mediator that can regulate α-synuclein-induced Ca^2+^ imbalance and cell apoptosis. In addition, parkin (the pathogenic gene for autosomal recessive juvenile PD) is partially located in the *trans-*Golgi network (Kubo et al., 2001). An increased expression of S-nitrosylated-parkin and p53 is simultaneously found in brain tissues of patients with PD. Sunico et al. (2013) further found that, in a mouse model of pesticide-induced PD, S-nitrosylation of parkin reduced its activity as a repressor of p53 gene expression, resulting in p53 upregulation, and p53 upregulation triggers neuronal apoptosis. These results suggest that p53-mediated neuronal apoptosis caused by S-nitrosylation of parkin is involved in the pathophysiology of PD.

### Hereditary Sensory and Autonomic Neuropathy Type II

Hereditary sensory and autonomic neuropathy type II (HSAN II) is a hereditary, degenerative, peripheral nervous system disease characterized by the progressive loss of peripheral sensory nerve function (Rotthier et al., 2012). Patients with HSAN II usually have progressive sensory and autonomic dysfunction that results in reduced sensitivity to pain, temperature, and touch (Arain and Chand, 2015). FAM134B is a newly identified *cis-*Golgi protein. The mutation of FAM134B directly causes HSAN II (Kurth et al., 2009; Falcao de Campos et al., 2019). Kurth et al. (2009) further found that FAM134B knockdown led to *cis-*Golgi compartment shrinkage that triggered the apoptosis of primary dorsal root ganglion neurons; this may be related to the neurotrophic factor transport disorder induced by FAM134B mutation. These results reveal that Golgi dysfunction is an important cause of neurogenetic diseases.

### Herpes Simplex Virus Encephalitis

Herpes simplex virus encephalitis (HSE) caused by HSV-1 infection is the most common sporadic encephalitis worldwide (Piret and Boivin, 2020). DeBiasi et al. (2002) found apoptotic neurons and glial cells in the brain tissue sections of patients with acute HSE. The GA exhibit a disordered and diffused cytoplasmic distribution in HSV-1-infected primary neurons (Martin et al., 2017). In addition, blood–brain barrier disruption is an important pathological mechanism for HSE development (Liu et al., 2019). Recently, He et al. (2020) found that HSV-1-induced blood–brain barrier damage involves apoptosis that is associated with GM130-mediated Golgi stress. The expression of GM130 is downregulated and accompanied by GA fragmentation in brain microvascular endothelial cells infected with HSV-1. The downregulation of GM130 promotes caspase cascade pathway activation. Eventually, activated caspases downregulate the tight junction proteins occludin and claudin 5, which implies the disrupted barrier function of endothelial cells. Interestingly, GM130 downregulation is partially caspase dependent (He et al., 2020). Moreover, apoptosis can inhibit HSV-1 proliferation in cells to a certain extent (Nguyen and Blaho, 2006), which has a certain positive significance for HSE. In short, the interaction between GA and apoptosis is closely related to the pathological mechanism of HSE.

### Ischemic Stroke

Stroke is the second most common cause of death worldwide, and ischemic stroke is the most common type of stroke (Wang et al., 2016). Cerebral ischemia–reperfusion injury is one of the important pathological mechanisms of ischemic stroke. P115 cleavage, Golgi fragmentation, and apoptosis have been observed *in vitro* in a cerebral ischemia–reperfusion model (Zhong et al., 2015). GOLPH3 is upregulated in a rat model of ischemic stroke (You et al., 2014). Li et al. further found that GOLPH3 not only acts as a Golgi stress sensor, owing to its rapid upregulation during oxidative stress, but also triggers Golgi stress and transmits a specific Golgi stress signal to downstream effectors. Stress-induced GOLPH3 upregulation leads to Golgi fragmentation and apoptosis (Li T. et al., 2016). Recently, Ran et al. (2007) discovered a 165-kDa protein induced by cerebral ischemia that was named ischemia-induced Golgi protein 165 (IIGP165) because of its localization in the GA. The Akt phosphorylation of IIGP165 prevents apoptotic cell death (Ran et al., 2007).

## Discussion

Multiple studies provide ample evidence that the structure and the function of the GA are damaged by various mechanisms during apoptosis. However, in the face of different apoptotic situations, the Golgi can inhibit or promote apoptosis through different Golgi-related proteins. Golgi-related apoptosis is involved in the pathological mechanisms of various neurological diseases. In the future, the Golgi may be a potential therapeutic target for apoptosis-related neurological diseases. However, the exact mechanisms and specific relationships between GA and apoptosis in numerous interactions must still be studied. Whether the Golgi is upstream or downstream of apoptosis in specific diseases should be studied. In addition, the crosstalk of apoptotic signals between the GA and the other subcellular organelles also requires investigation.

## Author Contributions

QH carried out the literature review and drafted the manuscript. HL, SD, and XC helped in drafting the manuscript. DL and XJ conceived, designed, and coordinated the study. WZ and WL contributed to and finalized the draft. All the authors read and approved the final manuscript.

## Conflict of Interest

The authors declare that the research was conducted in the absence of any commercial or financial relationships that could be construed as a potential conflict of interest.

## Abbreviations

AD: Alzheimer’s disease
ALS: amyotrophic lateral sclerosis
BI-1: Bax inhibitor 1
BIR: baculovirus-IAP repeat
Cdk5: cyclin-dependent kinase 5
COPI: coat protein complex I
CTF: C-terminal fragment
ER: endoplasmic reticulum
FasL: Fas ligand
FH2: formin homology 2
FHDC1: formin homology 2 domain protein 1
GA: Golgi apparatus
GAAP: Golgi anti-apoptotic protein
HSAN II: hereditary sensory and autonomic neuropathy type II
HSE: *Herpes simplex* virus encephalitis
IIGP165: ischemia-induced Golgi protein 165
IP3R: inositol-1,4,5-trisphosphate receptor
NAD^+^: nicotinamide adenine dinucleotide
p75NTR: p75 neurotrophin receptor
PD: Parkinson’s disease
PUMA: p53 upregulated modulator of apoptosis
TNFR: tumor necrosis factor receptor
UBC: ubiquitin-conjugating enzymes
UPR: unfolded protein response.
